# Physiological mechanisms of dehydration tolerance contribute to the invasion potential of *Ceratitis capitata* (Wiedemann) (Diptera: Tephritidae) relative to its less widely distributed congeners

**DOI:** 10.1186/s12983-016-0147-z

**Published:** 2016-03-31

**Authors:** Christopher W. Weldon, Leigh Boardman, Danica Marlin, John S. Terblanche

**Affiliations:** Department of Zoology and Entomology, University of Pretoria, Private Bag X20, Hatfield, 0028 South Africa; Centre for Invasion Biology, Department of Conservation Ecology and Entomology, Stellenbosch University, Private Bag X1, Matieland, 7602 South Africa; Present address: Department of Entomology and Nematology, University of Florida, PO Box 110620, Gainesville, FL 32611-0620 USA; Present address: School of Animal, Plant and Environmental Sciences, University of the Witwatersrand, 1 Jan Smuts Avenue, Braamfontein, 2000 South Africa

**Keywords:** Desiccation resistance, Water loss, Dehydration tolerance, Starvation, Body composition

## Abstract

**Background:**

The Mediterranean fruit fly, *Ceratitis capitata* (Wiedemann) (Diptera: Tephritidae) is a highly invasive species now with an almost cosmopolitan distribution. Two other damaging, polyphagous and closely-related species, the marula fruit fly, *Ceratitis cosyra* (Walker), and the Natal fly, *Ceratitis rosa* Karsch, are not established outside of sub-Saharan Africa. In this study, adult water balance traits and nutritional body composition were measured in all three species at different temperatures and levels of relative humidity to determine whether tolerance of water stress may partially explain their distribution.

**Results:**

Adult *C. capitata* exhibited higher desiccation resistance than *C. rosa* but not *C. cosyra*. Desiccation resistance of *C. capitata* was associated with lower rates of water loss under hot and dry conditions, higher dehydration tolerance, and higher lipid reserves that were catabolised during water stress. In comparison with *C. capitata*, *C. cosyra* and *C. rosa* lost water at significantly higher rates under hot, dry conditions, and did not catabolise lipids or other sources of metabolic water during water stress.

**Conclusions:**

These results suggest that adult physiological traits permitting higher tolerance of water stress play a role in the success of *C. capitata*, particularly relative to *C. rosa*. The distribution of *C. cosyra* is likely determined by the interaction of temperature with water stress, as well as the availability of suitable hosts for larval development.

**Electronic supplementary material:**

The online version of this article (doi:10.1186/s12983-016-0147-z) contains supplementary material, which is available to authorized users.

## Background

Environmental stress resistance, and a species ability to adapt to novel or variable environments, is of central interest in biology as it contributes to niche partitioning and biogeographic patterns. Environmental adaptations have particular importance for invasion biology because the ability of invasive species to survive variable environmental conditions has been suggested as a key trait that contributes to their dispersal and potential to invade new habitats [1]. Invasive species are introduced species that cause negative impacts on the environment, human activities, or human health [1]; as such they are regarded as major global threats and their management is an international research priority. Invasive insects pose a particular threat because they can be highly mobile and have high reproductive rates [2]. As climate change increases global temperatures, the threat of invasive insect species will increase as tropical and subtropical insects expand their range into temperate areas [3]. In this context, the ability of insects to withstand desiccation is likely to contribute to their invasive potential because, as they move away from the wetter tropical and subtropical regions, they will be exposed to reduced rainfall and increasingly dry environments [3].

The genus *Ceratitis* (Diptera: Tephritidae) includes true fruit flies where females oviposit and larvae develop in ripening fruit. For this reason, some members of the genus, particularly those with wide host ranges, are notorious pests of commercial fruit production. Most *Ceratitis* species have an Afrotropical distribution [4], but those that are pests can also be highly invasive. The Mediterranean fruit fly, *Ceratitis capitata* (Wiedemann) is a case in point [5, 6]. Historical records of spread, and analyses of biochemical and molecular markers (e.g., see [7, 8]) suggest that the species originated in central eastern Africa. In association with the growth and development of the international fresh fruit trade [5, 6], *C. capitata* has been introduced to, established successfully in, and expanded its range throughout many tropical, subtropical, and mild temperate habitats of the world [9–11]. The current, almost cosmopolitan distribution in suitable climates (excluding only central and eastern Asia; [12]) and highly polyphagous use of fruit hosts [reared from over 150 plant species in Africa alone; [13] has led to this species being considered the most economically damaging pest of horticulture in the world because of direct crop losses, pre- and postharvest control costs, and limited access or loss of access to fly-free export markets (e.g., see [14]). In contrast, two other damaging, polyphagous fruit flies from the same genus, the marula fruit fly, *Ceratitis cosyra* (Walker), and the Natal fly, *Ceratitis rosa* Karsch, have not become established outside of sub-Saharan Africa. *Ceratitis cosyra* has a much smaller host range than *C. capitata* (mostly recorded from the fruit of the indigenous marula, *Sclerocarya birrea* (A. Rich.) Hochst., or mango, *Mangifera indica* L., as well as other Anacardiaceae and Annonaceae), but its distribution stretches across central and eastern Africa [15]. *Ceratitis rosa* has a similar host range (in terms of species used and breadth of species) to *C. capitata*, but it is found only on the eastern side of Africa from Kenya, south in to South Africa, as well as some Indian Ocean islands where it has been introduced [15].

The successful invasion of *C. capitata* has been attributed to a range of factors. *Ceratitis capitata* is generally regarded as an *r*-selected species with life history traits that favour colonisation, such as small size, early reproduction, high growth rates, and efficient dispersal. It does not, however, have high competitive abilities, being displaced to ecological refuges where bioclimatic conditions do not favour competitively dominant species [16]. Field [17] and laboratory data [18–21] suggest that *C. capitata* can tolerate a wide range of temperatures, and that they outperform *C. rosa* at high temperatures in terms of adult critical thermal maximum and survival under semi-field conditions. Tolerance of water loss by *C. capitata*, or other *Ceratitis* species, has not been clearly established, but Duyck P-F, David P and Quilici S [17] provide data that indicate that *C. capitata* pupae are resistant to desiccation although intolerant of flooding. This differed from pupae of *C. rosa*, which were intolerant of desiccation but were unaffected by immersion in water for at least one hour. Data on the thermal limits or desiccation resistance of any life stage of *C. cosyra* are yet to be published, so the role of abiotic variables in determining their distribution is unclear.

There are only three possible, non-exclusive mechanisms for increased desiccation resistance at the organismal level: (1) increase water storage, (2) restrict water loss, or (3) increase tolerance to water loss [22]. These physiological mechanisms have all been reported in desiccation resistant species and populations, and have emerged in laboratory selection experiments. For example, different desiccation resistant lines of *Drosophila melanogaster* Meigen have reduced water permeability of the cuticle as a consequence of altered lipid composition, increased hemolymph volume, higher extracellular carbohydrate storage that increases hemolymph osmolality [23], and elevated tolerance to water loss and lipid storage [24]. Water content and dehydration tolerance are key to desiccation resistance of southern African keratin beetles [25, 26]. Desiccation resistance can also be achieved through reduced rates of respiration; *Drosophila* species from dry environments are less active, have lower metabolic rates, and exhibit cyclic CO_2_ emission patterns that may reduce water loss caused by transpiration [27]. In adult tephritid flies, improvement in desiccation resistance has been associated with increased body size [28, 29], which is associated with higher water content [30]. There is also evidence that some populations of the Queensland fruit fly, *Bactrocera tryoni* (Froggatt), tolerate higher levels of water loss than others before death [30], although the molecular basis for this observation remains unknown.

This study determined the desiccation resistance of the adults of three tephritid fly species, *C. capitata*, *C. cosyra* and *C. rosa*, with the particular aim of establishing whether tolerance of water stress may contribute to the invasive success of *C. capitata*. Potential mechanisms underpinning desiccation resistance in the tested species were also investigated. To do so, desiccation resistance of each species (scored as survival) was assessed at four different levels of relative humidity (0, 33, 75 and 100 %) at both 25 and 30 °C. A temperature of 25 °C is optimal for many life history traits in *C. capitata* and *C. rosa*, whereas long-term exposure to 30 °C impairs key fitness traits in both species [18]. Water content prior to the survival assay, water loss rates under the imposed conditions, water remaining at death after the assay (dehydration tolerance), and body composition prior to and after dehydration (0 % relative humidity) or starvation (100 % relative humidity) were also measured to identify whether they reflected patterns of desiccation resistance. It was predicted that desiccation resistance of *C. capitata* would be higher than in other species, and this would result from either higher body water content, lower water loss rates, or higher tolerance to loss of water.

## Results

### Initial condition

**Fig. 1:**
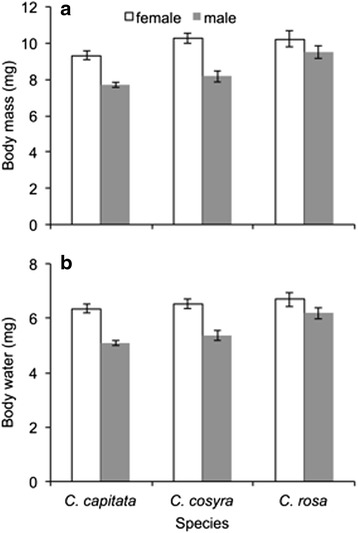
Body mass (**a**) and mass-adjusted body water (**b**) of adult females and males of *Ceratitis capitata*, *c. cosyra* and *c. rosa*. Body water was predicted from a general linear model that included body mass as a covariate

Body mass of the three *Ceratitis* species was not significantly different from each other (Table 1). The range of body mass for *C. capitata* was 6.5–11.6 mg, and that for *C. cosyra* and *C. rosa* was 6.5–12.7 mg and 5.4–13.2 mg, respectively. In all species, females were significantly heavier than males (Fig. 1a). Body mass significantly predicted water content in all species (Table 1), with water content being higher in heavier flies (Additional file 1: Table S1). Mass-adjusted water content was significantly affected by species, sex, and the interaction of species and sex (Table 1). In *C. capitata* and *C. cosyra*, females had a higher water content than males, but the water content of females and males was similar in *C. rosa* (Fig. 1b).

**Table 1:** General linear model for the effect of species and sex on body mass and body water of three *Ceratitis* species

Dependent variable	SS	df	F	*P*
Body mass (mg)				
Intercept	1739.11	1	913.320	<0.001
Species	11.54	2	3.031	0.052
Sex	27.39	1	14.384	**<0.001**
Species × Sex	9.68	2	2.541	0.083
Residuals	217.07	114		
Body water (mg)				
Intercept	1.118	1	11.516	<0.001
Species	1.525	2	7.858	**<0.001**
Sex	0.634	1	6.531	**0.012**
Body mass	79.312	1	817.171	**<0.001**
Species × Sex	0.724	2	3.731	**0.027**
Residuals	10.967	113		

Body mass was included as a covariate in the model for body water. Significant effects (*P* < 0.05) are indicated by bold type

### Desiccation resistance and dehydration tolerance

Survival of flies during desiccation resistance assays was significantly affected by the main effects of species, sex, temperature, relative humidity and initial mass (Additional file 2: Table S2). Overall, *C. capitata* and *C. cosyra* survived significantly longer during survival assays than *C. rosa*. However, complex significant interactions between species, sex, temperature and relative humidity were also found in the minimal adequate parametric survival model (Additional file 2: Table S2). For this reason, separate survival analyses were conducted for each species to explore the variation caused by the measured explanatory variables.

**Fig. 2:**
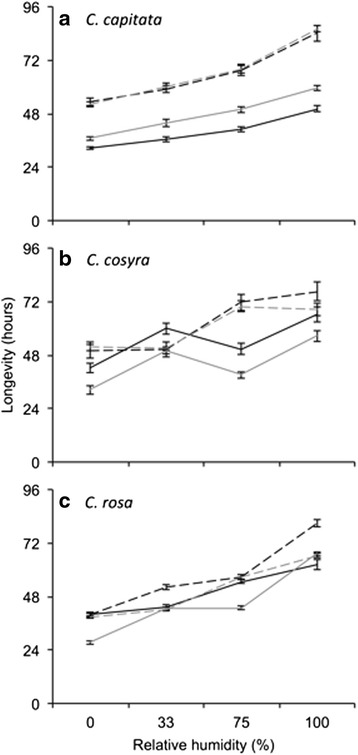
Longevity of *Ceratitis capitata* (**a**), *C. cosyra* (**b**) and *C. rosa* (**c**) during an assay where individual females and males were starved at different temperatures and levels of relative humidity. Longevity was predicted from a parametric survival regression that included initial body mass as a covariate. Line colour represents the sexes: black = female, grey = male. Line style represents temperature: dashed = 25 °C, solid = 30 °C

Survival time of *C. capitata* was significantly affected by the main effects of sex, temperature, relative humidity and initial mass (Table 2). Males survived longer than females, survival was shorter at 30 °C relative to 25 °C, and survival time improved with increasing relative humidity (Fig. 2a), while accounting for increased survival in individuals with higher initial mass (coefficient estimate = 0.306, s.e. = 0.089, *t* = 3.427, *p* < 0.001).

**Table 2:** Analysis of deviance table for the final fitted parametric survival model that describes desiccation resistance of three *Ceratitis* species with respect to sex, temperature (Temp) and relative humidity (RH)

Predictor	*χ* ^2^	df	*P*
*C. capitata*			
Sex	9.091	1	**0.003**
Temp	57.463	1	**<0.001**
RH	57.639	3	**<0.001**
Initial mass	16.668	1	**<0.001**
Sex × Temp	2.969	1	0.085
*C. cosyra*			
Sex	7.250	1	**0.007**
Temp	38.796	1	**<0.001**
RH	26.596	3	**<0.001**
Initial mass	31.884	1	**<0.001**
Temp × RH	18.694	3	**<0.001**
*C. rosa*			
Sex	0.002	1	0.965
Temp	1.852	1	0.174
RH	51.066	3	**<0.001**
Initial mass	15.480	1	**<0.001**
Sex × Temp	3.724	1	0.054
Sex × RH	5.126	3	0.163
Temp × RH	4.847	3	0.183
Sex × Temp × RH	17.247	3	**<0.001**

Initial body mass was included as a covariate in the model. Data were fitted to a Weibull hazard function. The analysis of deviance table was constructed using Type III likelihood ratio tests. Significant effects (*P* < 0.05) are indicated by bold type

Within *C. cosyra*, the main effects of sex, temperature, relative humidity and initial mass were also significant predictors of survival, as well as the interaction of temperature and relative humidity (Table 2). When accounting for increased survival time as initial mass increased (coefficient estimate = 0.490, s.e. = 0.056, *t* = 8.820, *p* < 0.001), female *C. cosyra* typically survived longer than males (Fig. 2b). When held at 25 °C, *C. cosyra* survived for approximately 50 h when held at 0 or 33 % relative humidity, but survived for approximately 70 h when relative humidity was higher. When held at 30 °C, survival time of *C. cosyra* was approximately 12 h shorter than at 25 °C, except at 33 % relative humidity (Fig. 2b).

Survival time of *C. rosa* was significantly affected by only the main effects of relative humidity and initial mass (Table 2). Again, survival was significantly improved by higher initial mass (coefficient estimate = 0.433, s.e. = 0.056, *t* = 7.745, *p* < 0.001). Survival generally increases with increasing relative humidity, but there was a significant interaction of sex and temperature with relative humidity (Table 2). The shortest survival time of *C. rosa* females and males was at 0 % relative humidity, but was particularly low among males at 30 °C where mean survival was only 27 h (Fig. 2c). Regardless of temperature, female *C. rosa* survival at 0 % relative humidity was similar, at approximately 39 h. This contrasts with female survival at 100 % relative humidity, where females held at 25 °C survived 18 h longer than those held at 30 °C.

**Fig. 3:**
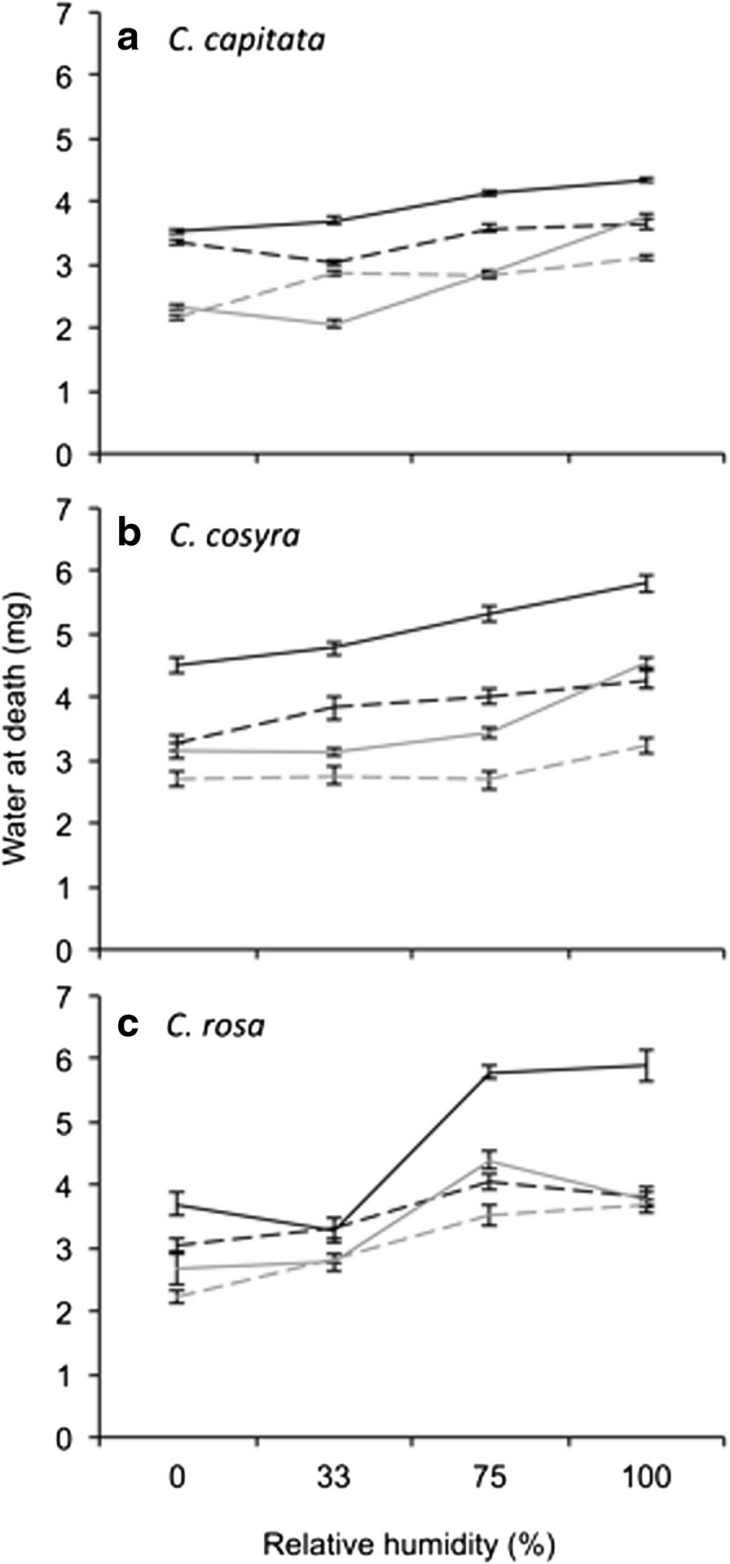
Water remaining at death (dehydration tolerance) in *Ceratitis capitata* (**a**), *C. cosyra* (**b**) and *C. rosa* (**c**) after having been starved at different temperatures and levels of relative humidity. Water at death was predicted from a general linear model that included an estimate of initial water content as a covariate to account for water available to be lost. Line colour represents the sexes: black = female, grey = male. Line style represents temperature: dashed = 25 °C, solid = 30 °C

Watercontent at death of flies subjected to the desiccation resistance assay, an indicator of dehydration tolerance, was significantly predicted by estimated body water (Additional file 3: Table S3), with water at death positively associated with increasing estimated body water (coefficient estimate = 0.429, s.e. = 0.036, *t* = 12.012, *p* < 0.001). When taking this covariate into account, there was a significant four-way interaction effect of species, sex, temperature and relative humidity on water at death (Additional file 3: Table S3). In separate analyses for each species (Table 3), water remaining at death of flies was also significantly positively associated with estimated body water (*C. capitata*: coefficient estimate = 0.306, s.e. = 0.089, t = 3.427, p < 0.001; *C. cosyra*: coefficient estimate = 0.490, s.e. = 0.056, *t* = 8.820, *p* < 0.001; *C. rosa*: coefficient estimate = 0.433, s.e. = 0.056, *t* = 7.745, *p* < 0.001). Within *C. capitata*, water at death was significantly affected by the main effect of sex, but also the three-way interaction of sex, temperature and relative humidity (Table 3). Overall, female *C. capitata* contained more water at death (i.e., were less dehydration tolerant) than males (Fig. 3a). When held at 30 °C, female *C. capitata* contained more water at death than those held at 25 °C. However, in male *C. capitata*, there was no consistent effect of temperature on water at death at different levels of relative humidity. Water at death of *C. cosyra* was significantly affected by the main effect of relative humidity, and the interactions of sex and relative humidity, and temperature and relative humidity (Table 3). Female *C. cosyra* were less dehydration tolerant than males in unsaturated air, but at 100 % relative humidity, water at death in males sharply increased (Fig. 3b). Water at death in *C. cosyra* stressed at 25 °C increased more gradually with increasing relative humidity than those stressed at 30 °C (Fig. 3b). Water at death in *C. rosa* was also significantly affected by the main effect of relative humidity, and the interaction of temperature and relative humidity (Table 3). In *C. rosa*, water at death increased significantly with increasing relative humidity, but there was a sharp increase in water at death from 33 to 75 and 100 % relative humidity when flies were held at 30 °C (Fig. 3c). Sex of stressed flies did not significantly affect dehydration tolerance of *C. rosa* (Table 3).

**Table 3:** Effects of sex, temperature (Temp) and relative humidity (RH) on the water at death ( dehydration tolerance) of three *Ceratitis* species estimated using general linear models

Dependent variable	SS	df	F	*P*
*C. capitata*				
Intercept	1.948	1	5.740	0.018
Sex	1.859	1	5.478	**0.021**
Temp	0.177	1	0.521	0.471
RH	1.974	3	1.939	0.126
Estimated body water	3.985	1	11.742	**<0.001**
Sex × Temp	0.002	1	0.007	0.935
Sex × RH	2.256	3	2.216	0.089
Temp × RH	0.977	3	0.959	0.414
Sex × Temp × RH	3.657	3	3.592	**0.015**
Residuals	48.534	143		
*C. cosyra*				
Intercept	0.863	1	3.842	0.052
Sex	0.550	1	2.447	0.120
Temp	0.039	1	0.172	0.679
RH	3.624	3	5.376	**0.002**
Estimated body water	18.392	1	81.849	**<0.001**
Sex × Temp	0.855	1	3.807	0.053
Sex × RH	2.446	3	3.628	**0.015**
Temp × RH	4.753	3	7.051	**<0.001**
Residuals	32.583	145		
*C. rosa*				
Intercept	0.649	1	1.164	0.282
Sex	1.622	1	2.909	0.090
Temp	0.108	1	0.193	0.661
RH	6.446	3	3.855	**0.011**
Estimated body water	33.444	1	59.991	**<0.001**
Sex × Temp	0.346	1	0.621	0.432
Sex × RH	0.463	3	0.277	0.842
Temp × RH	10.212	3	6.106	**<0.001**
Sex × Temp × RH	3.476	3	2.079	0.106
Residuals	79.720	143		

Estimated body water (determined from initial body mass using the equations in Additional file 1: Table S1) was included as a covariate. Significant effects (*P* < 0.05) are indicated by bold type

### Water loss rate

**Fig. 4:**
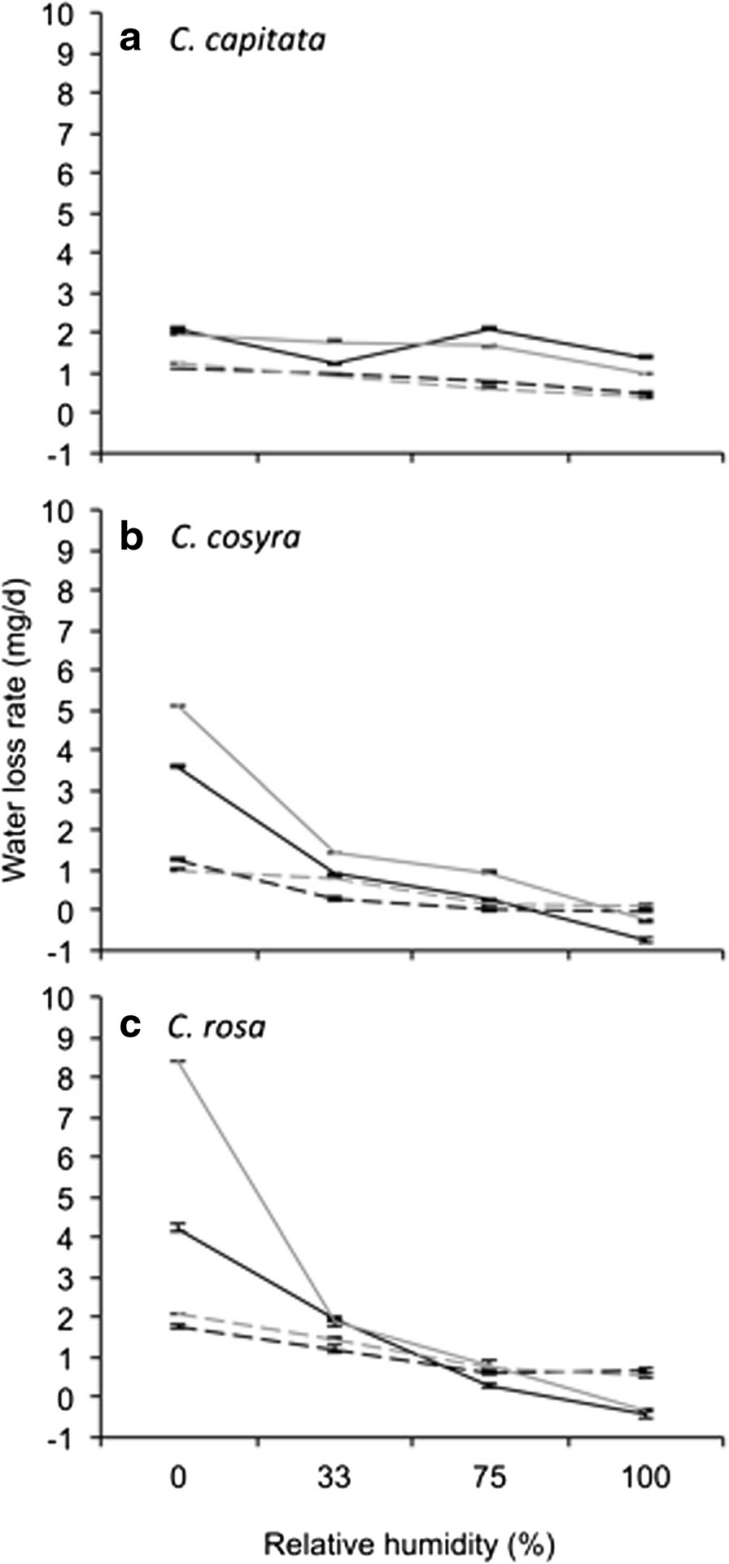
Water loss rate of *Ceratitis capitata* (**a**), *C. cosyra* (**b**) and *C. rosa* (**c**) starved at different temperatures and levels of relative humidity for 24 h. Water loss rate was predicted from a general linear model that included initial body mass as a covariate. Line colour represents the sexes: black = female, grey = male. Line style represents temperature: dashed = 25 °C, solid = 30 °C

Water loss over a period of 24 h was affected by the four-way interaction of species, sex, temperature and relative humidity, and by initial mass (Additional file 4: Table S4). Overall, *C. rosa* suffered significantly higher rates of water loss than *C. capitata* and *C. cosyra*, principally driven by very high rates of water loss when held at 30 °C and 0 % relative humidity (Fig. 4). When analysed separately, water loss rates of all three *Ceratitis* species were significantly affected by the main effects of temperature and relative humidity, and the interaction thereof (Table 4). For all three species, water loss rate increased with temperature, and declined as relative humidity increased (Fig. 4). However, the form of these responses differed among the species. When held at 25 °C, water loss by *C. capitata* declined significantly from low levels at 0 % relative humidity to 0.45 mg/day at 100 % relative humidity (Fig. 4a). Water loss by *C. capitata* held at 30 °C did not follow a distinct decline with increasing relative humidity, instead fluctuating between 1.23 and 2.06 mg/day across the tested range of relative humidity. In *C. cosyra* and *C. rosa*, water loss rate declined as relative humidity increased, to the extent that both species gained water in saturated air (Fig. 4b, c). Water loss rate in *C. cosyra* and *C. rosa* was particularly pronounced at 30 °C and 0 % relative humidity, being at least double, but up to four times greater, than water loss rate at 25 °C.

**Table 4:** Effects of sex, temperature (Temp) and relative humidity (RH) on the water loss rate (expressed per day) of three *Ceratitis* species estimated using general linear models

Dependent variable	SS	df	F	*P*
*C. capitata*				
Intercept	0.102	1	0.594	0.443
Sex	0.195	1	1.137	0.289
Temp	3.766	1	21.933	**<0.001**
RH	1.819	3	3.532	**0.018**
Initial mass	0.378	1	2.203	0.141
Sex × Temp	0.066	1	0.382	0.538
Sex × RH	0.205	3	0.398	0.755
Temp × RH	2.467	3	4.790	**0.004**
Sex × Temp × RH	1.013	3	1.966	0.125
Residuals	15.453	90		
*C. cosyra*				
Intercept	5.080	1	22.170	<0.001
Sex	0.455	1	1.987	0.162
Temp	17.719	1	77.331	**<0.001**
RH	8.016	3	11.661	**<0.001**
Initial mass	0.756	1	3.299	0.073
Sex × Temp	1.916	1	8.362	**0.005**
Sex × RH	1.184	3	1.722	0.168
Temp × RH	18.055	3	26.266	**<0.001**
Sex × Temp × RH	1.5291	3	2.225	0.091
Residuals	21.080	92		
*C. rosa*				
Intercept	1.583	1	5.649	0.020
Sex	0.336	1	1.200	0.276
Temp	9.373	1	33.450	**<0.001**
RH	6.551	3	7.793	**<0.001**
Initial mass	2.948	1	10.520	**0.002**
Sex × Temp	10.582	1	37.766	**<0.001**
Sex × RH	0.230	3	0.274	0.844
Temp × RH	17.383	3	20.678	**<0.001**
Sex × Temp × RH	8.723	3	10.377	**<0.001**
Residuals	25.499	91		

Initial body mass was included as a covariate. Water loss rate was estimated by subtracting body water after stress for a period of 24-h from estimated initial body water (determined from initial body mass using the equations in Additional file 1: Table S1). Significant effects (*P* < 0.05) are indicated by bold type

Water loss rate of *C. cosyra* and *C. rosa* was also affected by the significant interaction of sex with temperature. In both cases, males held at 30 °C had higher rates of water loss than females, but this difference was not evident at 25 °C (Fig. 4b, c). In *C. rosa*, there was an additional significant interaction of sex, temperature and relative humidity, which reflected a pronounced difference in water loss rate between females and males at 30 °C and 0 % relative humidity, with males losing water at a rate twice that of females (Fig. 4c). Water loss rate in *C. capitata* and *C. cosyra* was not associated with initial mass, but it was affected by initial mass in *C. rosa* (Table 4), with water loss rate of *C. rosa* becoming higher (coefficient estimate = 0.101, s.e. = 0.031, *t* = 3.243, *p* = 0.002) as initial mass increased.

### Carbohydrate, glycogen, lipid and protein content

The body composition of the three *Ceratitis* species that were starved at 0 or 100 % relative humidity varied significantly from flies that were not stressed (Table 5). Estimates of total carbohydrate (coefficient estimate = 0.092, s.e. = 0.037, *t* = 2.518, *p* = 0.016), glycogen (coefficient estimate = 0.009, s.e. = 0.002, *t* = 3.996, *p* < 0.001), total lipid (coefficient estimate = 0.011, s.e. = 0.003 *t* = 2.925, *p* = 0.006) and total protein content (coefficient estimate = 0.093, s.e. = 0.028, *t* = 3.338, *p* < 0.002) were all positively associated with initial mass. When taking initial mass into account, total carbohydrate content of the three *Ceratitis* species was significantly affected only by treatment (Table 5), with initial carbohydrate content of all three species being higher than when starved for 24 h at 0 or 100 % relative humidity (Table 6). Glycogen content was significantly affected by the main effect of treatment, and the interaction of species and treatment (Table 5). Relative to initial levels, glycogen content was much higher in starved *C. capitata* held at 100 % relative humidity. The same increase was not evident in other species. Lipid content was also affected by the main effect of treatment, and the significant interaction of species and treatment (Table 5). In this case, lipid content of *C. capitata* held at 0 % relative humidity declined far more than those held at 100 % relative humidity from levels estimated in control flies (Table 6). Lipid content did not vary between species in control flies, nor were there significant differences between treatments within *C. cosyra* or *C. rosa*. Protein content was significantly different between species (Table 5), with *C. rosa* containing significantly more protein than *C. capitata*, with *C. cosyra* intermediate between the other species (Table 6). In no species did protein content vary with treatment.

**Table 5:** Minimal adequate models explaining the effects of species, sex^a^, and treatment on the body composition of three *Ceratitis* species estimated using general linear models

Dependent variable	SS	df	F	*P*
Carbohydrates				
Intercept	15.104	1	42.484	<0.001
Treatment	8.977	2	12.625	**<0.001**
Initial mass	2.254	1	6.338	**0.016**
Residuals	14.932	42		
Glycogen				
Intercept	0.001	1	1.010	0.321
Species	0.005	2	3.140	0.053
Treatment	0.012	2	7.323	**0.002**
Initial mass	0.013	1	15.968	**<0.001**
Species × Treatment	0.010	4	2.868	**0.034**
Residuals	0.036	43		
Lipids				
Intercept	0.008	1	5.982	0.019
Species	0.007	2	2.734	0.077
Sex	0.002	1	1.733	0.195
Treatment	0.037	2	13.891	**<0.001**
Initial mass	0.011	1	8.554	**0.006**
Species × Sex	0.007	2	2.758	0.075
Species × Treatment	0.015	4	2.907	**0.033**
Residuals	0.054	41		
Proteins				
Intercept	0.013	1	0.131	0.719
Species	1.393	2	7.068	**0.002**
Sex	0.096	1	0.974	0.329
Treatment	0.301	2	1.527	0.229
Initial mass	1.098	1	11.145	**0.002**
Species × Sex	0.540	2	2.740	0.076
Sex × Treatment	0.478	2	2.422	0.101
Residuals	4.2372	43		

Initial body mass was included as a covariate to account for body size. Significant effects (*P* < 0.05) are indicated by bold type

^a^Sex was not included in the initial model for carbohydrates due to low sample size for males in some treatments

**Table 6:** Body composition of three species of *Ceratitis* prior to and after a 24-h period of starvation at 25 °C and 0 % or 100 % relative humidity

Species	*C. capitata*			*C. cosyra*			*C. rosa*		
Treatment	Control	100 % r.h.	0 % r.h.	Control	100 % r.h.	0 % r.h.	Control	100 % r.h.	0 % r.h.
Female	(*n* = 4)	(*n* = 3)	(*n* = 3)	(*n* = 7)	(*n* = 7)	(*n* = 7)	(*n* = 6)	(*n* = 7)	(*n* = 4)
Fresh mass	10.53 ± 0.63	13.60 ± 0.21	8.23 ± 0.54	11.20 ± 0.52	10.34 ± 0.70	9.59 ± 0.29	11.67 ± 0.66	16.61 ± 1.37	11.18 ± 0.41
Water	6.00 ± 0.68	9.67 ± 0.20	5.07 ± 0.38	7.27 ± 0.42	7.26 ± 0.50	6.11 ± 0.28	7.50 ± 0.53	12.03 ± 1.83	7.45 ± 0.35
Dry mass	4.53 ± 0.80	3.93 ± 0.07	3.17 ± 0.18	3.96 ± 0.36	3.09 ± 0.24	3.47 ± 0.24	4.17 ± 0.40	4.59 ± 0.61	3.73 ± 0.17
Carbohydrates	0.273 ± 0.035	0.148 ± 0.046	0.153 ± 0.014	0.554 ± 0.158	0.237 ± 0.130	0.399 ± 0.265	0.401 ± 0.166	0.272 ± 0.108	0.236 ± 0.067
Glycogen	0.049 ± 0.007	0.153 ± 0.005	0.067 ± 0.023	0.093 ± 0.018	0.047 ± 0.015	0.055 ± 0.018	0.071 ± 0.015	0.075 ± 0.020	0.101 ± 0.008
Lipids	0.305 ± 0.019	0.255 ± 0.030	0.217 ± 0.016	0.228 ± 0.033	0.202 ± 0.028	0.217 ± 0.020	0.258 ± 0.014	0.252 ± 0.019	0.284 ± 0.023
Proteins	0.708 ± 0.060	0.999 ± 0.314	1.284 ± 0.201	1.147 ± 0.090	1.057 ± 0.133	1.225 ± 0.154	1.382 ± 0.205	1.356 ± 0.156	1.914 ± 0.046
Male	(*n* = 4)	(*n* = 3)	(*n* = 4)	(*n* = 6)	(*n* = 3)	(*n* = 3)	(*n* = 5)	(*n* = 6)	(*n* = 5)
Fresh mass	8.05 ± 0.31	15.57 ± 1.56	5.88 ± 0.19	9.02 ± 0.68	6.77 ± 0.55	6.87 ± 0.19	11.04 ± 0.86	11.50 ± 0.53	8.60 ± 0.48
Water	4.68 ± 0.35	12.10 ± 1.33	3.90 ± 0.27	6.52 ± 0.66	4.87 ± 0.64	4.53 ± 0.27	6.96 ± 0.56	7.95 ± 0.37	4.56 ± 0.31
Dry mass	3.38 ± 0.58	3.47 ± 0.28	1.98 ± 0.15	2.63 ± 0.28	1.90 ± 0.10	2.33 ± 0.12	4.08 ± 0.37	3.55 ± 0.49	4.04 ± 0.47
Carbohydrates	0.538 ± 0.138	0.135 ± 0.032	0.088 ± 0.006	0.206^a^	0.080 ± 0.017	0.298 ± 0.209	0.437^a^	0.220 ± 0.105	0.083^a^
Glycogen	0.029 ± 0.009	0.153 ± 0.005	0.052 ± 0.021	0.057 ± 0.019	0.023 ± 0.013	0.035 ± 0.012	0.090 ± 0.025	0.101 ± 0.023	0.075 ± 0.008
Lipids	0.256 ± 0.013	0.229 ± 0.016	0.150 ± 0.027	0.148 ± 0.018	0.154 ± 0.010	0.191 ± 0.010	0.225 ± 0.015	0.191 ± 0.019	0.218 ± 0.024
Proteins	0.769 ± 0.202	0.791 ± 0.240	0.696 ± 0.131	0.952 ± 0.130	1.065 ± 0.153	0.999 ± 0.052	1.019 ± 0.158	1.274 ± 0.141	1.207 ± 0.052

Values are the mean ± 1 s.e., in milligrams. Fresh mass of flies held at 100 % r.h. and 0 % r.h. is fly mass after starvation for 24 h under each environmental condition

^a^Sample size = 1

## Discussion

Adult *C. capitata* are not unique in their level of desiccation resistance in comparison with the other pest *Ceratitis* species included in this study. The desiccation resistance of adult *C. cosyra* was equivalent to that of *C. capitata*, despite not having become widely distributed in different parts of the world. However, adults of both *C. capitata* and *C. cosyra* were more desiccation resistant than *C. rosa*. These results align with those for the pupal stage of *C. capitata* and *C. rosa*, with the former resisting dehydration for longer than the latter [17], and adds to the growing literature supporting a physiological basis [19–21, 31] for the spatial partitioning of the two species at continental [15] and regional scales [32]. They also align with recent modelling that predicts the area of suitable habitat for *C. rosa* will decline precipitously as climate warming and drying continues in the future [33]. Given the relatively high desiccation resistance of *C. cosyra*, from the perspective of water relations it is perhaps not surprising that its distribution across central Africa and along the eastern side of the continent aligns with the tropical dry savannah climate zone [15]. What is currently unclear is the reason for *C. cosyra* being found only in the north-eastern provinces of South Africa, which is an important consideration due to its potential to spread in a changing climate. Average and maximum temperatures in South Africa have increased over the last 50 years and are predicted to continue to do so [34]. If the range of *C. cosyra* is delimited by temperature, it may affect more areas of the country as the climate changes. Considering the desiccation resistance of adults, the associated drying that is also predicted [34, 35] will likely be of little consequence for *C. cosyra*. However, as *C. cosyra* has relatively few known host plant species, usually utilising fruit of plants in the Anacardiaceae and Annonaceae, climate change may have little effect on their southern distribution unless these hosts are cultivated or southern populations adapt to utilise alternative hosts. The latter situation is not unlikely due to the well-studied cases of host race evolution in tephritid flies (e.g., [36–39]).

The mechanisms that have evolved to increase the desiccation resistance of *C. capitata* and *C. cosyra* are not consistent. The data suggest that the desiccation resistance of *C. capitata* is derived from relatively high dehydration tolerance and low rates of water loss that are unaffected by atmospheric vapour pressure. In addition, it seems likely that *C. capitata* metabolises lipid reserves during periods of water stress (but not during starvation). Lipid content of adult *C capitata* not subjected to any stress was also higher than that of the other species. Lipid content has sometimes been associated with improved desiccation resistance in *Drosophila* species [24], but in other studies on *Drosophila*, laboratory selection for desiccation resistance has led to reduced [40] or no change in [41] lipid content. Like *C. capitata*, the adults of the xeric-adapted tsetse, *Glossina pallidipes* Austen, exhibited a marked decline in lipid content when starved at 0 % relative humidity relative to those stressed at 99 % relative humidity (at 25 °C; [42]). Lipid catabolism has been noted during desiccation in desert beetles, leading to the production of metabolic water [26, 43–45], but beetles and other insects from more mesic habitats, where water is freely available, have not been found to exhibit this capability [26, 42, 46].

Mechanisms for the relatively high desiccation resistance of *C. cosyra* are not as evident from the assays performed in this study. Water loss rate of *C. cosyra* was lower than in *C. rosa*, but still higher than in *C. capitata*, especially at high vapour pressure deficits. It remains to be determined how *C. capitata* and *C. cosyra* achieve lower rates of water loss than *C. rosa*; detailed study of water loss via the cuticle, spiracles and excretion, and cuticular lipid composition, are required to establish this. Neither *C. capitata* nor *C. cosyra* had more body water, nor were they more tolerant of dehydration than *C. rosa*. On the contrary, *C. capitata* had lower body water than *C. rosa* despite being more desiccation resistant. This result is surprising because in other tephritid flies [28, 30] as well as other model insect systems [23, 26, 42, 47] enhanced desiccation resistance has been associated with higher body water content. Under saturated conditions, water at death increased and water loss rates became negative in *C. cosyra* and *C. rosa*. It is highly likely that this was caused by the drinking of condensation by starved flies, but may have also resulted from gains in water content from metabolism or passive sorption of water from the atmosphere [48].

Temperature was an important determinant of how adults of the tested *Ceratitis* species were able to tolerate differences in humidity. In all species, lower survival times and higher rates of water loss, and lower dehydration tolerance were observed at higher temperatures when flies were exposed to unsaturated atmospheric conditions. These results differ somewhat from those reported for the adults of four *Glossina* species, where water loss rates in relation to relative humidity and temperature were not consistent across the species [42]. Higher rates of water loss at higher temperatures in *C. cosyra* and *C. rosa* may be associated with lower melting points of waterproofing cuticular lipids, resulting in higher rates of cuticular water loss [23]. Males of *C. cosyra* are smaller than females, so rates of water loss after having been adjusted for initial mass of males should be higher assuming that they have a lower surface area-volume ratio. Another possibility not accounted for in the design of this study is that male activity within tubes may have been higher than that of females, leading to higher water loss via the respiratory pathway. Female and male *B. tryoni* [49] and *Drosophila melanogaster* Meigen [50] of the same age exhibit different patterns of spontaneous activity, which has been interpreted to reflect differences in life history and physiological requirements of the sexes.

Unlike the other tested species, when held at 100 % relative humidity, water loss rates of *C. capitata* were higher at 30 °C than at the benign temperature of 25 °C. This result, together with lower variation in desiccation resistance and dehydration tolerance in response to relative humidity at 25 °C, suggests that some of the invasive potential of *C. capitata* does not stem from a more plastic response to desiccating conditions, as would be predicted by the greater flexibility hypothesis [51]. Baker HG [51] proposed that invasive species have greater plasticity than indigenous ones, which enables their survival under novel conditions. In an explicit test of this hypothesis, Chown et al. [3] found for springtails on Marion Island that flexibility in the response of indigenous and invasive species to acclimation did not differ, but rather acclimation at warmer temperatures led to improved desiccation resistance at both test temperatures. An ability to maintain fitness under stressful conditions was found in the invasive slug, *Arion lusitanicus* (Mabille), where high fecundity under conditions of limited food and high temperatures were retained relative to an indigenous congener [52]. In contrast to this “Jack-of-all-trades” phenotype [53], *C. capitata* appears to have a consistent basal resistance to water stress under benign thermal conditions relative to conspecifics. Using the terminology of Richards CL, Bossdorf O, Muth NZ, Gurevitch J and Pigliucci M [53], *C. capitata* is rather a “Master of some” with regard to water stress, and better able to increase fitness in favourable environments. This response under stressful conditions is important for the establishment of *C. capitata* in new areas, and links well with models that predict its potential distribution. A model for world-wide seasonal suitability for *C. capitata* developed by Szyniszewska AM and Tatem AJ [12] was principally driven by minimum land surface temperature, followed by maximum land surface temperature, elevation, then maximum rainfall. Assuming no negative biotic interactions with other tephritid species, our results indicate that *C. capitata* will largely be able to withstand water stress as long as the thermal environment in a newly colonised area is suitable.

Across the *Ceratitis* species tested, adult dehydration tolerance was lower in females. This result is similar to those reported for the Mexican fruit fly, *Anastrepha ludens* Loew [28] and *B. tryoni* [29, 30]. It has been proposed that reduced dehydration tolerance in female tephritid flies is a result of gravid females not having access to the pool of water contained within developing eggs [28, 30], and this is also a likely scenario for the *Ceratitis* species studied here because they were tested at an age (10 days after adult emergence) when they are known to be sexually mature if fed a nutritious diet [54]. Given this, it is surprising that there was no effect of sex on the lipid or protein content of the three *Ceratitis* species. It is not unrealistic to expect that gravid females should have a higher lipid content of these nutrients due to their investment in eggs. Inability to detect this effect may reflect the differential investment of nutrients for different life history strategies between the sexes, as found in *C. capitata* [55].

## Conclusion

In conclusion, tolerance of water stress by adults is likely to play a role in the invasive success of *C. capitata*. They exhibit relatively high levels of desiccation resistance that is associated with high tolerance of water loss, low rates of water loss, storage of lipids, and the ability to catabolise lipids during periods of water stress, which are particularly beneficial at benign temperatures. Relative to *C. rosa*, these properties would enable survival of adult *C. capitata* in drier environments so that they are able to reproduce and become established when conditions become more favourable. However, tolerance of water stress alone is not sufficient to explain why *C. cosyra* has not become as widespread as *C. capitata*. The interaction of temperature with water stress, as well as the influence of host availability are likely important in proscribing the distribution of *C. cosyra*.

## Methods

### Source of flies and culture maintenance

Flies of each species tested in this experiment, *C. capitata*, *C. cosyra* and *C. rosa*, were initially sourced from large, outbred laboratory populations maintained by Citrus Research International, Nelspruit, South Africa. The cultures of each species are maintained at CRI in high numbers and have been maintained in the laboratory for nearly 200 generations. The cultures are regularly supplemented with wild flies caught during summer to minimise inbreeding (wild males are paired with laboratory females) and are held indoors under buffered, but variable temperatures (25 ± 4 °C) with natural lighting. While the tested cultures have been held in the laboratory for a long period of time, these cultures have been used to test differences in the thermal tolerance of *C. capitata* and *C. rosa* [56], and as in those studies, water balance traits should represent baseline genotypic differences between the species. Eggs laid by females are placed on a carrot-based medium in which larvae develop. Pupae were transported to the University of Pretoria by road in a cooler box. On arrival at the University of Pretoria, pupae of each species were placed into separate plastic cages (volume 5 L) and kept in a climate room at 25 ± 1 °C until they emerged. On adult emergence, approximately 300 adults of each species (mixed sexes) were transferred to separate 5-L plastic cages furnished with sugar and yeast extract powder (Biolab, Merck, Wadeville, Gauteng, South Africa) in separate dishes for food, and a container of water-soaked cotton wool.

Ten days after adult emergence, oviposition dishes were placed in each cage into which females could lay eggs. Oviposition dishes were circular plastic cups (Volume Injection Products, East London, Eastern Cape, South Africa) with a volume of 125 mL that contained one sheet of tissue paper that had been sprayed with distilled water. To the damp tissue, 2 mL of guava concentrate (Elvin Food and Beverages, East London, Eastern Cape, South Africa) was applied with a 3 mL graduated plastic Pasteur pipette (Delta Lab, Barcelona, Spain). The opening of the plastic container was covered with a double layer of laboratory film (Parafilm M, Bemis Flexible Packaging, Neenah, WI, USA) that was then pierced 10–15 times with an entomological pin. Eggs laid by female *C. capitata*, *C. cosyra* and *C. rosa* into the oviposition dishes were rinsed, separately, from the laboratory film and container with distilled water from a wash bottle into a flat-bottomed glass evaporating dish. Eggs in water were concentrated in the evaporating dish by gently swirling the contents into the centre. Eggs were then transferred with a plastic Pasteur pipette to 500 mL of the same carrot-based medium used by Citrus Research International for larval development (no more than 600 eggs/500 mL). To avoid contamination of each culture, the evaporating dish was washed with boiling water between each *Ceratitis* species and a new plastic Pasteur pipette was used. Containers of larval rearing medium with eggs were placed in clear plastic trays with ventilated lids that were lined with a layer of washed, dry beach sand to a depth of 10 mm. At the end of the third larval instar, larvae ‘hopped’ from the larval rearing medium and entered the pupal phase in the layer of sand. The sand was sifted to recover the pupae, which were then returned to the culture or directed to experiments.

*Ceratitis capitata*, *C. cosyra* and *C. rosa* pupae used for experiments (~300 of each species) were placed in separate 5 L plastic cages with sources of food (sugar, hydrolysed yeast powder) and water. This was a low cage stocking density (surface area of ~6.5 cm^2^ per fly, not taking into consideration additional area from food and water containers within the cage) to prevent potential negative interactions at higher density [57]. On adult emergence, all cages were then inserted into clear plastic bags that were sealed to maintain high humidity (>90 %) and ensure that flies were well hydrated, and kept in a laboratory maintained at 25 ± 1 °C. Two cohorts of each species were required to complete the study, with one cohort per test temperature (25 or 30 °C).

### Initial condition

At 10 days after adult emergence, 20 flies of each species (10 females and 10 males) were placed in pre-weighed 2 mL microcentrifuge tubes of known weight. The initial mass of each fly was measured (to 0.1 mg; AR0640, Ohaus Corporation, Pine Brook, NJ, USA) before storage in a freezer at −20 °C. At a later time, these flies were dried in a fan-forced laboratory oven at 60 °C for 96 h and weighed on a microbalance (to 0.001 mg; CPA2P, Sartorius AG, Goettingen, Germany) to determine body water and dry mass. Initial mass of these flies was determined using an analytical balance rather than a microbalance to minimise the time required to weigh them and thereby reduce measurement bias introduced by handling time. Ordinary least squares regression was used to relate initial mass to initial body water content for each species, sex and cohort (test temperature). Linear regression provided the best fit for all relationships when compared with non-linear functions. The resulting regression equations (Additional file 1: Table S1) were used to estimate initial body water based on measurements of initial mass in subsequent experiments.

### Desiccation resistance and dehydration tolerance

At 10 days after adult emergence, 40 females and 40 males of each species were placed into individual, numbered, pre-weighed 2 mL microcentrifuge tubes pierced with 12 holes (approximate diameter = 1 mm). Flies in tubes were then weighed (to 0.1 mg) and initial mass of each fly was calculated by subtracting tube mass. Ten females and 10 males of each species were then allocated to one of four humidity treatments: 0 %, 33 %, 75 % and 100 %. Relative humidity was manipulated by placing plastic cups with 200 mL of silica gel, saturated magnesium chloride solution, saturated sodium chloride solution or deionised water, respectively, in clear plastic airtight boxes (volume 4.5 L; Ref 02 1474, Hobby Life ®, Demirel Plastik, Turkey). Actual values for relative humidity recorded with Thermochron iButtons (DS1921G, Maxim Integrated, San Jose, CA, USA) in each treatment were: 0 %: −1.29 ± 0.14 % (at 25.12 ± 0.01 °C); 33 %: 33.38 ± 0.06 % (at 24.95 ± 0.01 °C); 75 %: 75.55 ± 0.11 % (at 25.24 ± 0.01 °C); 100 %: 99.27 ± 0.16 % (at 25.11 ± 0.01 °C). All airtight boxes were maintained at 25 °C for the duration of the experiment by placing them in an incubator. The experiment was then repeated at 30 °C using a second cohort of flies. Actual values for relative humidity recorded in the airtight boxes during these treatments were: 0 %: −0.59 ± 0.11 % (at 29.05 ± 0.02 °C); 33 %: 33.69 ± 0.04 % (at 29.41 ± 0.01 °C); 75 %: 76.17 ± 0.07 % (at 30.11 ± 0.01 °C); 100 %: 100.70 ± 0.07 % (at 29.01 ± 0.02 °C).

Desiccation resistance was defined as the time that elapsed before death. All tubes were checked for mortality every 3 h by viewing them through the clear plastic top of the airtight boxes. Dead flies were removed from their tubes, weighed (to 0.01 mg), returned to their tubes and stored in a freezer at −20 °C. Removal of tubes required that the airtight boxes be unsealed, but iButton recordings indicated a rapid return to experimental conditions after this disturbance. Later, all flies were dried and weighed as described above to determine body water and dry mass at death. Dehydration tolerance, defined as water loss before death, was calculated by subtracting body water at death from estimated initial body water (following the relationships in Additional file 1: Table S1).

### Water loss rate

At 10 days after adult emergence, 30 females and 30 males of each species were placed into pierced microcentrifuge tubes and their initial mass was determined as above. Fifteen flies were then allocated to one of four humidity treatments, as above. After 24 h at 25 °C, flies were removed from each humidity treatment, weighed in their vials (to 0.1 mg) to calculate weight loss, and then placed in a freezer at −20 °C. Flies were then dried and weighed, as above, to determine body water and dry mass after 24 h. Water loss rate (mg day^−1^) was calculated by subtracting body water after 24 h from estimated initial body water (following the relationships in Additional file 1: Table S1). The experiment was then repeated at 30 °C.

### Carbohydrate, glycogen, lipid and protein content assays

Adult flies were obtained for the determination of lipid, free sugar, glycogen and protein content by repeating the desiccation resistance and dehydration tolerance experiments. This was necessary because values for carbohydrate and protein content were negatively affected by oven-drying of flies at 60 °C. Levels of relative humidity achieved during these repeats were measured with Thermochron iButtons (0 %: −0.43 ± 0.50 % at 24.34 ± 0.70 °C; 100 %: 73.16 ± 2.86 % at 23.88 ± 0.47 °C). For each species, control flies were weighed on an electronic microbalance (MS104S, Mettler Toledo, Greifensee, Switzerland) and immediately frozen at −80 °C, and additional flies were subjected to either 0 % or 100 % relative humidity (25 °C) for 24 h. Flies were weighed before and after each treatment (to 0.1 mg) before being frozen. Flies were freeze-dried to obtain dry mass and thereby water content, and refrozen at −80 °C.

Protein, carbohydrate, glycogen and total lipid contents were obtained from each individual insect for each species (aiming to assay a minimum of 6 for each control/treatment: 3 male, 3 female) using colourimetric methods developed by Bradford MM [58] and van Handel [59–61] and modified into a microplate format by Foray V, Pelisson P-F, Bel-Venner M-C, Desouhant E, Venner S, Menu F, Giron D and Rey B [62]. Where modifications to the methods described by Foray V, Pelisson P-F, Bel-Venner M-C, Desouhant E, Venner S, Menu F, Giron D and Rey B [62] were made, the procedure is described in detail below. Sugar and glycogen samples were run in duplicate on the plate, while protein and lipid samples were run in triplicate. Absorbance values were averaged to obtain one absorbance value per biological sample. In all cases, a flat-bottom 96-well borosilicate microplate (730.009QG, Hellma Analytics, Germany) was used, and absorbance was read using a microplate reader (BioTek Powerwave 340) and Gen5^TM^ v.1.05.11 software (both BioTek Instruments, Inc.). Each species was assayed on a separate day and standards were used to compare between species.

Whole flies were homogenized in 180 μL lysis buffer (100 mM KH_2_PO_4_, 1 mM DTT, 1 mM EDTA, pH 7.4) for 20 s at 25 Hz using a mixer mill (MM 400, Retsch, Germany). The protein content was determined by gently centrifuging the homogenate and adding 1.5 μL of the supernatant or 2 mg/ml albumin standard (23209, Thermo Scientific) dissolved in lysis buffer to each well. 250 μL of Bradford micro-assay reagent (B6916, Sigma) was added to each well and incubated at room temperature for 20 min before absorbance was read at 595 nm. Lipids and soluble carbohydrates were extracted by adding 20 μL 20 % sodium sulfate, 4.5 μL of lysis buffer and 1500 μL chloroform methanol (1:2 v/v) solution to the homogenate. The homogenate was vortexed and centrifuged at 180 *g* for 15 min (4 °C). The supernatant containing the carbohydrates and lipids was transferred to a new tube, while the glycogen pellet was kept. Glycogen was extracted from the pellet following Yuval B, Kaspi R, Shloush S and Warburg MS [63]. The pellet was washed twice with 400 μL 80 % methanol and centrifuged at 16 000 g for 5 min (4 °C). After removing the supernatant, 250 μL dH_2_O was added to the pellet and heated at 70 °C for 5 min. 1 mL fresh anthrone reagent (319899, Sigma; 1.42 g/L) was added to 200 μL of sample or standard (1 mg/ml glucose (D+) dissolved in dH_2_O, SAAR2676020EM, Merck, South Africa) and incubated at 90 °C for 10 min. The reaction was stopped by cooling the sample on ice before 200 μL of sample was added to each well and the absorbance was read at 625 nm. Total water soluble carbohydrates were determined following Foray V, Pelisson P-F, Bel-Venner M-C, Desouhant E, Venner S, Menu F, Giron D and Rey B [62]. Fresh anthrone reagent was used to induce a colourimetric reaction measured at 625 nm. The standard from the glycogen step was used to quantify the amount of sugar present see [64]. The total lipid content was determined using vanillin reagent (V1104, Sigma) with triolein (44895, Sigma) as the standard, following Foray V, Pelisson P-F, Bel-Venner M-C, Desouhant E, Venner S, Menu F, Giron D and Rey B [62]. Absorbance was measured spectrophotometrically at 525 nm.

Absorbance values were converted to mg amounts using standard curves. The amount of each nutrient present in each total fly was estimated. All estimates of total water soluble carbohydrates higher than 1 mg were deleted, as were estimates of glycogen that were greater than 0.2 mg. In the case of total carbohydrates, this led to the exclusion of two *C. capitata*, fifteen *C. cosyra*, and eighteen *C. rosa* from data analysis.

### Data analyses

All data analyses were performed using the R v. 3.1.2 statistical environment (R Development Core Team, 2012). Body mass and body water were compared between species using linear models. Predictor variables in each model were species, sex and their interaction. In the model for body water, body mass was included as a covariate. Model-checking plots were inspected and revealed that the models did not violate assumptions of constancy of variance and normality of errors. Least squares means of the response variable for these and all models described below were determined using the ‘predict’ function in R with covariates at their mean.

Parametric survival regression with Weibull age-specific hazards was performed using the ‘survival’ library of R to determine the effect of species, sex, temperature and relative humidity on desiccation resistance. Initial body mass was included as a covariate in the model to account for differences in the pool of available water in each individual or size effects on body composition. The minimal adequate model was determined based on Akaike’s information criterion (AIC) using the ‘step’ function in R, which involves the step-wise deletion of the least influential parameters from the model. Because significant effects of species or an interaction with species were found for desiccation resistance, models were run separately for each species with sex, temperature and relative humidity as predictor variables.

The effects of species, sex, temperature and relative humidity and their interactions on dehydration tolerance and water loss rate were determined using linear models. In the case of dehydration tolerance, the dependent variable was body water at death, which was related to estimated body water by inclusion of the latter as a covariate in the model. In the model for water loss rate, initial mass was included as a covariate because body mass has been previously shown to correlate strongly with water loss [30]. In both cases, there was a significant four-way interaction between species, sex, temperature and relative humidity, so the models were run separately for each species before being simplified based on comparison of AIC values. Flies that died during the water loss rate assay were excluded from analysis. These models did not violate the assumptions of constancy of variance and normality of errors.

Carbohydrate, glycogen, lipid and protein content in flies not subjected to stress, and either desiccated or starved were compared using linear models. Predictor variables in the initial model for carbohydrate content included only species and treatment (i.e., initial condition, 24 h at 0 % relative humidity, 24 h at 100 % relative humidity), their interaction, and initial mass. Sex was not included in this model due to there being too few reliable estimates of carbohydrate content from each sex in the three species. Values for carbohydrate content were log-transformed to improve model fit. The initial linear models for glycogen, lipid and protein content included sex and its interactions with species and treatment, with initial mass as a covariate. Initial mass rather than dry mass was used as a covariate in these models because we wished to establish how body composition varied relative to the initial condition of each fly rather than its condition after a period of stress. Models were simplified based on comparison of AIC values.

## Additional files

Additional file 1:
**Table S1.** Linear regression for the relationship between body mass (mg) and body water content (mg) for cohorts of three *Ceratitis* species that were subsequently tested for desiccation resistance and water loss rate at two temperatures. The equation for each relationship was used to estimate initial body water content from initial body mass for flies subjected to desiccation and water loss rate assays. (DOC 41 kb) Additional file 2:
**Table S2.** Analysis of deviance table for the final fitted parametric survival model that describes desiccation resistance of three *Ceratitis* species with respect to species, sex, temperature (Temp) and relative humidity (RH). Initial body mass was included as a covariate in the model. Data were fitted to a Weibull hazard function. Type III likelihood ratio tests were used to construct the analysis of deviance table. Significant effects (*P* < 0.05) are indicated by bold type. (DOC 34 kb) Additional file 3:
**Table S3.** General linear model for the relationship between species, sex, temperature (Temp) and relative humidity (RH) on the dehydration tolerance of three *Ceratitis* species. Estimated body water (determined from initial body mass using the equations in Table S1) was included as a covariate. Significant effects (*P* < 0.05) are indicated by bold type. (DOC 37 kb) Additional file 4:
**Table S4.** General linear model for the relationship between species, sex, temperature (Temp) and relative humidity (RH) on water loss rate of three *Ceratitis* species. Initial mass was included as a covariate. Significant effects (*P* < 0.05) are indicated by bold type. (DOC 37 kb)
