# Supplementary material for: Physiological mechanisms of dehydration tolerance contribute to the invasion potential of Ceratitis capitata (Wiedemann) (Diptera: Tephritidae) relative to its less widely distributed congeners
Source: Front Zool. 2016 Mar 31;13:15. doi: 10.1186/s12983-016-0147-z (PMC4815119; doi:10.1186/s12983-016-0147-z)
Supplement: Additional file 1: — Table S1. Linear regression for the relationship between body mass (mg) and body water content (mg) for cohorts of three Ceratitis species that were subsequently tested for desiccation resistance and water loss rate at two temperatures. The equation for each relationship was used to estimate initial body water content from initial body mass for flies subjected to desiccation and water loss rate assays. (DOC 41 kb) [file 12983_2016_147_MOESM1_ESM.doc]

**Table S1.** Linear regression for the relationship between body mass (mg) and body water content (mg) for cohorts of three *Ceratitis* species that were subsequently tested for desiccation resistance and water loss rate at two temperatures. The equation for each relationship was used to estimate initial body water content from initial body mass for flies subjected to desiccation and water loss rate assays.

| Cohort | Equation | R^2^ | F_(1,8)_ | P-value |
| --- | --- | --- | --- | --- |
| 25˚C |  |  |  |  |
| *Ceratitis capitata* |  |  |  |  |
| Female | y = 0.665x + 0.150 | 0.978 | 354.921 | <0.001 |
| Male | y = 0.799x - 1.031 | 0.816 | 35.399 | <0.001 |
| *Ceratitis cosyra* |  |  |  |  |
| Female | y = 0.677x - 0.402 | 0.742 | 23.000 | 0.001 |
| Male | y = 0.030x + 4.535 | 0.002 | 0.019 | 0.895 |
| *Ceratitis rosa* |  |  |  |  |
| Female | y = 0.673x + 0.065 | 0.892 | 8.658 | <0.001 |
| Male | y = 0.668x + 0.039 | 0.896 | 69.049 | <0.001 |
| 30˚C |  |  |  |  |
| *Ceratitis capitata* |  |  |  |  |
| Female | y = 0.593x + 0.849 | 0.921 | 93.438 | <0.001 |
| Male | y = 0.654x + 0.084 | 0.855 | 47.285 | <0.001 |
| *Ceratitis cosyra* |  |  |  |  |
| Female | y = 0.702x - 0.722 | 0.836 | 50.798 | <0.001 |
| Male | y = 0.506x + 1.418 | 0.902 | 55.050 | <0.001 |
| *Ceratitis rosa* |  |  |  |  |
| Female | y = 0.554 + 0.868 | 0.920 | 92.034 | <0.001 |
| Male | y = 0.648 - 0.149x | 0.962 | 205.236 | <0.001 |
