# Supplementary material for: Physiological mechanisms of dehydration tolerance contribute to the invasion potential of Ceratitis capitata (Wiedemann) (Diptera: Tephritidae) relative to its less widely distributed congeners
Source: Front Zool. 2016 Mar 31;13:15. doi: 10.1186/s12983-016-0147-z (PMC4815119; doi:10.1186/s12983-016-0147-z)
Supplement: Additional file 2: — Table S2. Analysis of deviance table for the final fitted parametric survival model that describes desiccation resistance of three Ceratitis species with respect to species, sex, temperature (Temp) and relative humidity (RH). Initial body mass was included as a covariate in the model. Data were fitted to a Weibull hazard function. Type III likelihood ratio tests were used to construct the analysis of deviance table. Significant effects (P < 0.05) are indicated by bold type. (DOC 34 kb) [file 12983_2016_147_MOESM2_ESM.doc]

**Table S2.** Analysis of deviance table for the final fitted parametric survival model that describes desiccation resistance of three *Ceratitis* species with respect to species, sex, temperature (Temp) and relative humidity (RH). Initial body mass was included as a covariate in the model. Data were fitted to a Weibull hazard function. Type III likelihood ratio tests were used to construct the analysis of deviance table. Significant effects (P < 0.05) are indicated by bold type.

| **Predictor** | **χ^2^** | **df** | **P** |
| --- | --- | --- | --- |
| Species | 9.045 | 2 | **0.011** |
| Sex | 5.003 | 1 | **0.025** |
| Temp | 20.697 | 1 | **<0.001** |
| RH | 29.490 | 3 | **<0.001** |
| Initial mass | 57.787 | 1 | **<0.001** |
| Species × Sex | 9.672 | 2 | **0.008** |
| Species × Temp | 1.295 | 2 | 0.523 |
| Species × RH | 11.835 | 6 | 0.066 |
| Sex × Temp | 0.361 | 1 | 0.548 |
| Sex × RH | 7.040 | 3 | 0.071 |
| Temp × RH | 5.574 | 3 | 0.134 |
| Species × Sex × Temp | 7.307 | 2 | **0.026** |
| Species × Temp × RH | 17.128 | 6 | **0.009** |
| Sex × Temp × RH | 11.146 | 3 | **0.011** |
