# Supplementary material for: Physiological mechanisms of dehydration tolerance contribute to the invasion potential of Ceratitis capitata (Wiedemann) (Diptera: Tephritidae) relative to its less widely distributed congeners
Source: Front Zool. 2016 Mar 31;13:15. doi: 10.1186/s12983-016-0147-z (PMC4815119; doi:10.1186/s12983-016-0147-z)
Supplement: Additional file 3: — Table S3. General linear model for the relationship between species, sex, temperature (Temp) and relative humidity (RH) on the dehydration tolerance of three Ceratitis species. Estimated body water (determined from initial body mass using the equations in Table S1) was included as a covariate. Significant effects (P < 0.05) are indicated by bold type. (DOC 37 kb) [file 12983_2016_147_MOESM3_ESM.doc]

**Table S3.** General linear model for the relationship between species, sex, temperature (Temp) and relative humidity (RH) on the dehydration tolerance of three *Ceratitis* species. Estimated body water (determined from initial body mass using the equations in Table S1) was included as a covariate. Significant effects (P < 0.05) are indicated by bold type.

| **Dependent variable** | **SS** | **df** | **F** | **P** |
| --- | --- | --- | --- | --- |
| Intercept | 1.810 | 1 | 4.8233 | 0.029 |
| Species | 1.576 | 2 | 2.100 | 0.124 |
| Sex | 1.190 | 1 | 3.171 | 0.076 |
| Temp | 0.183 | 1 | 0.489 | 0.485 |
| RH | 1.851 | 3 | 1.644 | 0.179 |
| Estimated body water | 54.145 | 1 | 144.276 | **<0.001** |
| Species × Sex | 0.117 | 2 | 0.156 | 0.856 |
| Species × Temp | 0.276 | 2 | 0.368 | 0.692 |
| Species × RH | 2.855 | 6 | 1.268 | 0.271 |
| Sex × Temp | 0.005 | 1 | 0.014 | 0.907 |
| Sex × RH | 2.089 | 3 | 1.856 | 0.137 |
| Temp × RH | 0.997 | 3 | 0.886 | 0.448 |
| Species × Sex × Temp | 0.525 | 2 | 0.699 | 0.498 |
| Species × Sex × RH | 2.275 | 6 | 1.012 | 0.418 |
| Species × Temp × RH | 4.791 | 6 | 2.128 | **0.049** |
| Sex × Temp × RH | 3.708 | 3 | 3.293 | **0.021** |
| Species × Sex × Temp × RH | 5.847 | 6 | 2.597 | **0.018** |
| Residuals | 161.372 | 430 |  |  |
