# Supplementary material for: Physiological mechanisms of dehydration tolerance contribute to the invasion potential of Ceratitis capitata (Wiedemann) (Diptera: Tephritidae) relative to its less widely distributed congeners
Source: Front Zool. 2016 Mar 31;13:15. doi: 10.1186/s12983-016-0147-z (PMC4815119; doi:10.1186/s12983-016-0147-z)
Supplement: Additional file 4: — Table S4. General linear model for the relationship between species, sex, temperature (Temp) and relative humidity (RH) on water loss rate of three Ceratitis species. Initial mass was included as a covariate. Significant effects (P < 0.05) are indicated by bold type. (DOC 37 kb) [file 12983_2016_147_MOESM4_ESM.doc]

**Table S4.** General linear model for the relationship between species, sex, temperature (Temp) and relative humidity (RH) on water loss rate of three *Ceratitis* species. Initial mass was included as a covariate. Significant effects (P < 0.05) are indicated by bold type.

| **Dependent variable** | **SS** | **df** | **F** | **P** |
| --- | --- | --- | --- | --- |
| Intercept | 1.680 | 1 | 7.114 | 0.008 |
| Species | 1.427 | 2 | 3.021 | **0.050** |
| Sex | 0.142 | 1 | 0.602 | 0.439 |
| Temp | 3.735 | 1 | 15.817 | **<0.001** |
| RH | 1.745 | 3 | 2.463 | 0.063 |
| Initial mass | 1.176 | 1 | 4.978 | **0.026** |
| Species × Sex | 0.475 | 2 | 1.006 | 0.367 |
| Species × Temp | 2.688 | 2 | 5.692 | **0.004** |
| Species × RH | 2.026 | 6 | 1.456 | 0.194 |
| Sex × Temp | 0.086 | 1 | 0.366 | 0.546 |
| Sex × RH | 0.195 | 3 | 0.275 | 0.844 |
| Temp × RH | 2.591 | 3 | 3.660 | **0.013** |
| Species × Sex × Temp | 8.539 | 2 | 18.081 | **<0.001** |
| Species × Sex × RH | 1.216 | 6 | 0.858 | 0.526 |
| Species × Temp × RH | 16.113 | 6 | 11.373 | **0.024** |
| Sex × Temp × RH | 0.987 | 3 | 1.393 | 0.245 |
| Species × Sex × Temp × RH | 8.093 | 6 | 5.712 | **<0.001** |
| Residuals | 64.938 | 275 |  |  |
